# Physical Activity During Pregnancy: Associations Between Levels and Types of Physical Activity and Low Back Pain–Related Disability in Portuguese Pregnant Women

**DOI:** 10.3390/ijerph23020245

**Published:** 2026-02-15

**Authors:** Isabel Teixeira, Paula Clara Santos, Clarinda Festas, Diana Bernardo

**Affiliations:** 1 Escola Superior de Saúde, Instituto Politécnico do Porto, 4249-015 Porto, Portugal; isabelrteixeiraft@gmail.com; 2 RISE-Health, Center for Translational Health and Medical Biotechnology Research (TBIO), ESS, Polytechnic of Porto, 400, 4200-072 Porto, Portugal; 3 Centro de Investigação em Reabilitação, Escola Superior de Saúde, Instituto Politécnico do Porto, 4249-015 Porto, Portugal; 4 Escola Superior de Saúde Fernando Pessoa (ESS-FP), 4200-253 Porto, Portugal; clarinda@ufp.edu.pt; 5 Instituto de Investigação, Inovação e Desenvolvimento Fernando Pessoa (I3ID-FP), Biomedical and Health Sciences (FP-BHS), 4249-004 Porto, Portugal; 6 RISE-Health, Fernando Pessoa Educational and Culture Foundation, 4200-253 Porto, Portugal; 7 Research Center for Active Living and Wellbeing (LiveWell), Instituto Politécnico de Bragança, 5300-253 Bragança, Portugal; 8 Escola Superior de Saúde, Instituto Politécnico de Bragança, 5300-253 Bragança, Portugal

**Keywords:** pregnant women, physical activity, sedentary lifestyle, low back pain, oswestry disability index

## Abstract

Low back pain (LBP) is one of the most prevalent musculoskeletal conditions during pregnancy and frequently impairs daily living activities and quality of life. The association between different levels and types of physical activity (PA) and LBP-related functional disability remains insufficiently explored. This study aimed to examine the association between PA levels and types and functional disability among pregnant women with LBP. A cross-sectional analytical study was conducted involving 192 Portuguese pregnant women. Data were collected through an online questionnaire comprising the Pregnancy Physical Activity Questionnaire and the Oswestry Disability Index (ODI). Most participants engaged predominantly in light-intensity or sedentary activity (69.1%), with minimal participation in vigorous activity (0.8%). Functional disability was generally mild (mean ODI = 11.5 ± 7.35); however, 42.2% of participants reported moderate disability and 11.0% severe disability. Advancing gestational age showed weak associations with increased domestic activity (r = 0.146, *p* = 0.044), decreased occupational activity (r = −0.295, *p* = 0.001), and higher functional disability scores (r = 0.142, *p* = 0.049). Parity was associated with differences in total PA levels (*p* = 0.005) and domestic activity (*p* = 0.001). Higher ODI scores were weakly associated with light-intensity and sedentary activity (r = 0.144, *p* = 0.047), whereas severe disability demonstrated a moderate association with sedentary behavior (r = 0.529, *p* = 0.014). Overall, lower levels of PA, particularly sedentary behavior, were weakly associated with higher LBP-related disability; however, the observed associations were generally weak and should be interpreted with caution considering the cross-sectional design.

## 1. Introduction

Low back pain (LBP) is defined as pain or discomfort located between the twelfth rib and the gluteal fold [1,2]. During pregnancy, gestational LBP is characterized either by ascending pain from the lumbar region or by pelvic/sacroiliac pain located at the lateral aspect of the fifth lumbar vertebra and extending distally. In some cases, it may be present as a combination of both musculoskeletal dysfunctions [3]. Pregnancy is associated with anatomical and physiological changes, and LBP is a recurrent event. The etiology of this condition during pregnancy remains unclear and is generally considered to be complex and multifactorial, involving biomechanical, vascular, and hormonal changes [4].

LBP is one of the most common musculoskeletal dysfunctions during pregnancy and has a significant impact on quality of life, healthcare costs, and productivity [5]. Reported prevalence during pregnancy ranges from 25% to 90%, with data indicating that approximately 50% of pregnant women experience gestational low back pain. Of these, 80% report that the pain interferes with their daily life activities (DLAs), and 10% are unable to perform their occupational tasks [5]. While LBP can be present from the first trimester, studies suggest that discomfort peaks during the third trimester [5,6]. Musculoskeletal structures seem to adapt their function in response to progressive uterine growth and hormonal fluctuations (e.g., relaxin). These are associated with an anterior shift in the center of mass in pregnant women and increased kinematic forces on the lumbar spine, thereby limiting postural adjustments [5,7].

According to the World Health Organization [8,9], PA is defined as any bodily movement produced by skeletal muscles that requires energy expenditure, including PA performed during work, play, household chores, travel, and recreational activities. Guidelines from the American College of Sports Medicine [9] and Canadian recommendations for PA during pregnancy [10] advise that pregnant women engage in at least 150 min of moderate-intensity physical activity per week. These recommendations also encourage sedentary women to begin PA during the preconception period. Regular PA during pregnancy is associated with maternal and fetal health and presents minimal risks [4]. Benefits have been associated with reduced risk of maternal complications such as gestational diabetes, hypertensive disorders, preeclampsia, postpartum depression, excessive weight gain, and postpartum weight retention [4,5,10] as well as improvements in overall functional status.

While PA is widely recommended during pregnancy, the evidence regarding its relationship with LBP remains inconsistent. Light- to moderate-intensity PA has been associated in some studies with improved functional capacity and reduced pain perception, whereas others report no clear association or even adverse effects when physical demands are prolonged or repetitive. In particular, sedentary behavior and sustained postures, frequently related to occupational activities, have been linked to increased musculoskeletal discomfort, including lower back pain.

Previous research conducted in Portugal has generally highlighted the relevance of examining how these activity patterns are associated with LBP-related functional disability [5].

Over the years, pregnancy has often been described as a “window of opportunity” for behavioral change and adoption of health-promoting practices, potentially optimizing the health status of both mother and baby. Commonly referred to as “a teachable moment,” it is characterized by increased individual motivation to modify risk behaviors. Improvements in these habits, during pregnancy, may lead to the adoption of healthier practices maintained into the postpartum period and may be associated with long-term health benefits, including for future pregnancies [11].

According to previous literature, the severity of LBP tends to be higher in later trimesters and represents one of the leading causes of work absenteeism and one of the most common musculoskeletal complaints among pregnant women [12]. LBP is also described as the fifth most frequent cause of emergency medical visits and is associated with a significant negative financial impact nowadays. Most women perceive the functional severity of LBP as a natural part of pregnancy and do not adopt preventive or corrective measures [12]. Current literature shows that individual factors influence not only PA levels but also the functional severity of LBP during pregnancy. Factors such as age, parity, educational level, gestational age, and pre-pregnancy body mass index (BMI) have been analyzed in several studies, demonstrating potential associations with PA levels and the characterization of LBP in pregnant women [13,14,15]. It is important to note that the distinction between different types of activity, such as domestic activities, occupational activities, and structured exercises, is often insufficiently addressed, with most studies focusing primarily on activity intensity. These activity domains may involve distinct biomechanical loads and postural demands, which have been discussed in the literature as potentially relevant for the severity of LBP during pregnancy. Domestic and occupational activities, although predominantly low intensity, may include prolonged standing, bending, or maintaining static positions, which have been associated with increased load on the spine during pregnancy.

However, studies conducted in Portugal remain scarce in addressing the relationship between PA levels and the severity of LBP during pregnancy. Moreover, few studies have simultaneously analyzed both PA intensity and PA type, which limit the understanding of their combined association on pain-related functional disability. This gap highlights the need for more comprehensive approaches that consider both dimensions when characterizing PA patterns in pregnant women.

Thus, this study aimed to examine the association between PA levels and types and LBP related functional disability in pregnant women, allowing for a more comprehensive characterization of activity patterns and their potential relationship with musculoskeletal health during pregnancy.

## 2. Materials and Methods

### 2.1. Study Design and Participants

This research follows an observational analytical and cross-sectional study design, using a quantitative methodology. The primary objective of this study was to characterize LBP-related disability, assessed with the Oswestry Disability Index (ODI-V2), and to examine its association with PA, including total PA volume, PA intensity, and domain-specific scores derived from the Pregnancy Physical Activity Questionnaire (PPAQ) The data were collected at a single moment through the administration of an online questionnaire, available via a QR code, accessible during nursing consultations at primary health care centers and at the hospital. In addition, the access link to the questionnaires was sent directly to the pregnant women by email. No a priori sample size calculation was performed. The sample size was determined by the number of eligible participants recruited during the data collection period. The final sample comprised 254 participants, which is comparable to or exceeds the sample sizes of similar studies, and provides sufficient precision for the planned statistical analyses.

The study population included pregnant women at different gestational trimesters, aged 18 or over, who were registered and followed in primary healthcare centers across mainland Portugal and the islands (ACeS), or at the São João University Hospital Center (CHUSJ). Eligibility criteria included: (a) pregnant women aged 18 or older; (b) registered and followed in ACeS or CHUSJ; (c) voluntary participation with signed informed consent; and (d) affirmative response to the question: “Do you currently have or have you had low back pain (pain in the lower back, which may or may not radiate to the leg) during this pregnancy?”. Exclusion criteria were: (a) lack of proficiency in Portuguese; (b) visual, or cognitive impairments that would prevent questionnaire completion; and (c) multiple pregnancies. Participation was contingent upon prior informed consent.

### 2.2. Assessment Tools

The online questionnaire comprised the following components: the PPAQ, the ODI—V2, and a sociodemographic questionnaire.

(a) Sociodemographic Questionnaire: This questionnaire was designed to collect personal, social, and lifestyle-related information, along with data on health status during pregnancy and obstetric history. Pre-pregnancy Body Mass Index (BMI) was calculated using self-reported height and pre-pregnancy weight using the Quetelet Index formula: BMI = weight (kg)/height (m)^2^.
(b) PPAQ—Pregnancy Physical Activity Questionnaire: It is a self-administered tool developed to assess PA levels during each trimester of pregnancy, measuring the frequency and duration of various activities, including multiple activity domains [16,17].

It includes 32 daily life activities (DLAs) such as household, occupational, and sports activities, referencing the prior month [16,17]. The Portuguese-translated and validated version makes the instrument reliable for data collection and ensures comparability of results [18].

Energy expenditure was calculated by multiplying time spent on each activity by its intensity, measured in METs (metabolic equivalent of task). Activities were classified according to intensity: sedentary (<1.5 METs), light (1.5–3.0 METs), moderate (3.0–6.0 METs), and vigorous (>6.0 METs) [17]. The Portuguese validated version of the PPAQ demonstrated good test–retest reliability, assessed using the intraclass correlation coefficient (ICC), with values ranging from 0.70 to 0.87 across different domains and an overall ICC of 0.77 [17]. Internal consistency in the current sample was assessed using Cronbach’s alpha and McDonald’s omega. For the PPAQ, α = 0.466 and ω = 0.561, indicating low internal consistency; therefore, the results are interpreted with caution.

(c) ODI V.02 – Oswestry Disability Index: is a widely used tool for evaluating functional disability related to LBP [19]. It has been used in various studies examining functional outcomes in pregnant populations [13,20,21]. The Portuguese version of the ODI 2.0, adapted for the Portuguese population, showed good internal consistency with a Cronbach’s alpha of 0.87, supporting its use for assessing functional disability related to LBP [22]. The questionnaire includes 10 items, each with six response options, addressing the impact of LBP on daily life activities: pain intensity, personal care, lifting, walking, sitting, standing, sleeping, sexual activity, social life, and recreation. Each item is scored from 0 to 5, where 0 indicates no dysfunction and 5 indicates maximum dysfunction (Likert scale) [22]. The maximum possible score is 50. If any item is unanswered, its score is subtracted from the total, and a percentage is calculated accordingly. Disability levels are interpreted as follows: 0–20%: minimal disability; 21–40%: moderate disability; 41–60%: severe disability; 61–80%: very severe disability; 81–100%: symptom exaggeration [23].

### 2.3. Statistical Analysis

Statistical analyses were conducted using IBM SPSS Statistics v.29.0. A significance level of 0.05 was adopted for descriptive and inferential statistics. Central tendency (mean) and dispersion (standard deviation) measures were used for sample characterization. Categorical variables were reported as frequencies and percentages.

Normality of continuous variables was assessed using the Shapiro–Wilk test. Several PA variables and the Oswestry Disability Index (ODI) showed deviations from normality (PPAQ intensity- moderate, vigorous; PPAQ activity-domestic and exercise; ODI Total), which is consistent with their expected asymmetric distribution. Pearson’s correlation coefficient was used for the main analyses to allow comparability with previous studies. To assess the robustness of the findings, Spearman’s rank correlation coefficients were additionally calculated as a sensitivity analysis. Spearman correlations showed consistent direction and magnitude compared with Pearson correlations, with no meaningful differences in statistical significance (Supplementary Material). As both approaches yielded comparable results, Pearson’s coefficients are presented in the main tables. Pearson’s correlation strength is classified as follows: |r| 0–0.3 = weak; |r| ≥ 0.3 and <0.6 = moderate; |r| ≥ 0.6 and <0.9 = strong; |r| ≥ 0.9 = very strong [24]. Given the sample size, Pearson’s correlation was considered sufficiently robust to moderate deviations from normality.

Given the distribution of several PA and disability variables, results should be interpreted with caution as indicators of association rather than precise estimates. No causal inferences were drawn.

For comparisons involving categorical (nominal or ordinal) variables, the Kruskal–Wallis test was applied.

## 3. Results

### 3.1. Study Population

**Figure 1:**
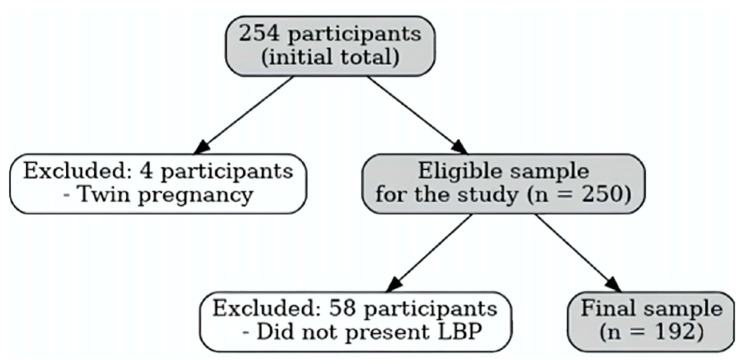
Sampling process.

The sampling and selection process is illustrated in Figure 1. Of the 254 participants who initially completed the data collection questionnaire, 4 were deemed ineligible due to multiple pregnancies. Subsequently, 58 pregnant women were excluded for not reporting LBP during pregnancy, resulting in a final valid sample of 192 participants.

#### Sample Characterization

The demographic characteristics of the sample are presented in Table 1. The final sample consisted of 192 pregnant women aged between 19 and 48, with a mean age of 31.7 (±5.13). Among them, 83.5% resided in the northern region of Portugal. Of the total sample, 79.1% of the women were in the third trimester of pregnancy, with a mean gestational age of 30.9 (±6.75) weeks. Regarding pre-pregnancy BMI, most participants were within the normal weight range (62%), while 35.4% had a BMI above the recommended threshold [24]. In terms of parity, nulliparous women represented the largest portion of the sample (62.6%), corresponding to 92 participants. The sample demonstrated a high level of education, with 87 women holding a university degree (45.3%), and 90.6% of the participants were employed.

### 3.2. Characterization of Physical Activity (PA)—Intensity and Type

The levels of PA are presented in Table 2. Regarding the intensity of PA, a tendency toward activities below 3 METs was observed, accounting for 69.1% of the participants’ daily metabolic expenditure. Light-intensity activity had a mean value of 95.4 (±45.56) METs·h·wk^−1^, representing the largest contribution to total energy expenditure. This was followed by moderate-intensity activity (30.1%) and sedentary activity (24.5%).). Vigorous intensity activity contributed minimally to total PA (0.8% %), with a mean of 1.8 (±3.87) METs·h·wk^−1^. In terms of PA types, 69.1% of metabolic expenditure was also largely attributable to household and occupational activities (52.3% and 16.8%, respectively), while only 4.9% of energy expenditure was related to sports activities. Additionally, inactivity represented 17.4% of total energy expenditure.

### 3.3. Characterization of Disability Caused by LBP (ODI)

Disability levels, according to the ODI score, are presented in Table 3. The mean disability score among participants was 11.5 (±7.35), which corresponds to a classification of mild disability. A higher percentage of pregnant women (43.2%) reported minimal disability. However, despite a slight difference, 81 pregnant women (42.2%) reported moderate disability related to the presence of LBP during pregnancy. Additionally, 11% of participants indicated severe disability in performing activities, in contrast with only six women (3.1%) who reported no associated disability.

Notably, 32.3% of participants reported the intensity of their pain as “mild” and 22.9% reported it as “moderate”. A total of 48 women (25%) reported an inability to lift heavy weights due to increased pain, and 35.4% of the sample reported being unable to remain seated for more than one hour due to worsening pain.

### 3.4. Correlation Between Physical Activity, Socio-Demographic Variables and ODI

The correlation analysis between sociodemographic data, disability, and PA is presented in Table 4. Statistical analysis indicated that age showed a weak negative association with moderate physical activity during pregnancy (r = −0.139), which did not reach statistical significance (*p* = 0.056), suggesting a borderline trend toward lower moderate activity with increasing age. This result indicates a possible trend toward decreased moderate PA as maternal age increases. Additionally, women with higher pre-pregnancy BMI showed a positive association with higher levels of vigorous PA (r = 0.157, *p* = 0.029).

Gestational age was associated with most variables. Domestic activity showed a slight increase as pregnancy progressed (r = 0.146, *p* = 0.044), while occupational activity was negatively associated with gestational age (r = −0.295, *p* = 0.001). Functional impairment tended to increase with advancing gestational age, showing a very weak, but statistically significant correlation (r = 0.142, *p* = 0.049). The results revealed statistically significant differences in total PA levels between parity groups (nulliparous vs. multiparous) (*p* = 0.005), indicating differences in the distribution of reported PA levels based on the number of previous births. Significant differences were also found in light (*p* = 0.001) and moderate (*p* = 0.045) intensity PA and in domestic PA (*p* = 0.001) between these groups. The functional severity index showed differences between parity groups; however, this association did not reach statistical significance (*p* = 0.052), suggesting only a borderline trend.

Regarding educational level, statistically significant differences were observed in sedentary PA levels across educational categories (*p* = 0.037), suggesting that higher educational achievement was associated with differences in sedentary behavior during pregnancy.

### 3.5. Correlation Analysis Between PA Levels and Total ODI Score

The correlation analysis between PA levels and ODI total score is presented in Table 5. Pregnant women with PA levels predominantly below 3 METs·h·wk^−1^, represented by the combined variables “light + sedentary” activity, showed a weak but statistically significant correlation with higher total ODI disability scores (r = 0.144, *p* = 0.047). There was a tendency for women with higher levels of domestic and occupational activity to show higher or lower disability scores, respectively, although these associations were not statistically significant (r = 0.103, *p* = 0.156 and r = −0.082, *p* = 0.256, respectively). Conversely, a significant positive association was observed between increased inactivity and higher functional disability (r = 0.192, *p* = 0.008). Finally, the correlation between total PA level (in METs) and total disability (ODI) was weak and not statistically significant, indicating no clear association (r = 0.027, *p* = 0.713).

#### Correlations Between Disability Levels (ODI) and PA Levels (PPAQ)

Correlations between disability levels, based on the total ODI score, and PA levels (PPAQ) are presented in Appendix A (Table A1). A trend was observed indicating that women with greater disability severity tended to report lower total PA levels; however, this correlation was weak and not statistically significant (r = 0.155, *p* = 0.050).

Correlations between ODI disability categories (mild, moderate, and severe) and PPAQ PA levels (sedentary, light, moderate, and vigorous), presented in Appendix A (Table A2), revealed a single significant relationship between severe disability and sedentary activity. This correlation was positive, moderate, and statistically significant (r = 0.529, *p* = 0.014). These findings suggest that severe levels of functional disability may be associated with lower PA levels among pregnant women.

Regarding the relationship between ODI disability levels and types of PA, shown in Appendix A (Table A3), pregnant women with severe disability exhibited higher energy expenditure in “physical inactivity”, with a moderate and statistically significant correlation (r = 0.498, *p* = 0.022). No other notable correlations were observed.

## 4. Discussion

The primary objective of this study was to examine PA levels and types among Portuguese pregnant women as well as the associations between PA practice and the severity of LBP during pregnancy.

The results indicate that PA patterns during pregnancy were associated with LBP related functional disability. Most pregnant women concentrated their energy expenditure in light-intensity activities, followed by sedentary activities, predominantly related to domestic and occupational tasks, which constituted the main sources of overall physical activity. These findings are generally consistent with previous studies [14,25,26,27]; however, given the cross-sectional design, it is not possible to determine the direction of this association, and it remains unclear whether higher disability is associated with increased sedentary behavior or whether greater sedentary behavior is associated with higher perceived disability. These findings should be interpreted with caution. In contrast, low engagement in sports related PA was observed, with a tendency toward reduced participation across gestational trimesters. Previous literature has similarly documented a decline in PA as pregnancy progresses [14,25,26,28]. However, given the cross-sectional design, these interpretations should be regarded as hypothetical, as the present study did not directly assess these factors.

Low participation in structured or sports-related activities may reflect several factors described in the literature, including the absence of structured exercise programs, perceived functional limitations, and misconceptions regarding the safety of more intense PA during pregnancy. Inconsistent or insufficient guidance from health professionals, particularly during the third trimester, has also been suggested as a possible contributing factor [16,25,26]. These explanations remain hypothetical within the context of the present study and warrant further investigation using longitudinal or qualitative approaches.

This study suggests that greater functional disability during pregnancy was weakly associated with higher levels of sedentary behavior and physical inactivity. Although these associations reached statistical significance, their magnitude was small; therefore, their clinical relevance remains uncertain. These findings should be interpreted with caution and considered exploratory in nature. The literature suggests that light PA contributes to lower daily metabolic expenditure [5,26,27,29], which may be insufficient to support optimal physical function during a period characterized by marked physiological and biomechanical changes [30].

Previous studies have reported that LBP prevalence and severity tend to peak during the third trimester and is a risk factor for postpartum continuation (approximately 40%) [31]. However, these findings should be interpreted with caution as the cross-sectional nature of the data precludes conclusions regarding progression or causality. Factors such as kinesiophobia, pain-related avoidance, fatigue, nausea, sleep disturbances, and lack of access to updated exercise protocols are frequently cited in the literature as potential contributors to lower PA levels during pregnancy [5,29,31,32,33]. However, these factors cannot be directly examined within the present study and are therefore presented as hypothesized explanations only.

Another finding was the association between gestational age and the functional disability index, indicating that higher gestational age was associated with higher disability scores. Given the borderline statistical significance of this association, these findings should be interpreted with caution. Nevertheless, this finding is consistent with previous studies [12,31]. Prior researches [5,31] argue that pre-pregnancy LBP and PA conditions during pregnancy are strong predictors for LBP development, with better functional status being associated with a lower incidence of LBP during pregnancy [34]. These contrasting findings highlight the complexity of parity-related patterns and suggest that individual and contextual factors may play an important role.

Norsyam et al. [12] further report that nulliparous women tend to experience more LBP compared to multiparous women, which may help explain the findings of the present study. The authors suggest that individual inexperience with biomechanical and hormonal changes, as well as subjective pain perception—potentially leading to an overestimation of discomfort—and the lower parental burden faced by nulliparous women compared to multiparous women, contribute to this paradoxical finding. However, the same study proposes that LBP is more disabling among multiparous women due to circadian rhythm alterations, decreased lumbopelvic mobility, and significant mechanical and structural changes in the abdominal core [12,27].

The sample in this study showed high levels of energy expenditure in occupational activities. Previous research suggests that less physically demanding job roles tend to remain unchanged during pregnancy compared to more demanding occupations. The influence of the “nesting effect” [5] is identified as a factor in the reduction of more strenuous PA, reinforcing the tendency toward low-energy domestic tasks associated with preparing for a new baby. However, current economic and social realities seem to influence the continued active participation of pregnant women in the workforce to maintain their financial independence and social status [26]. Nevertheless, these explanations are derived from previous literature and cannot be confirmed within the present cross-sectional design. Thus, they should be viewed as contextual considerations rather than established causal pathways.

According to other authors [14,25], women with higher occupational activity tend to have fewer children, whereas women with greater number of children tend to engage less in sports-related PA. A study published in 2025 reports that employed women have a 57.9% lower risk of sedentary behavior compared to unemployed pregnant women. Moreover, women with two or more children are nine times more likely to reach moderate-intensity PA levels, suggesting that parental responsibilities may be a key factor driving PA, as women are traditionally viewed as the primary family caregivers [26].

Studies also emphasize the importance of PA in general for health benefits; however, in cases of neuromusculoskeletal conditions such as LBP, a more structured and specific exercise plan, encompassing sports PA, may be beneficial. Pregnant women with LBP may benefit from guided exercise programs that include re-education and strengthening of lumbopelvic stabilizers. Core stability exercises have proven effective in controlling functional severity caused by LBP during pregnancy [35,36,37,38].

It was also noted that pregnant women with higher educational levels tend to exhibit higher sedentary behavior levels, although these findings partly diverge from those of previous researchers [3,26], where primiparous women with higher education more frequently met recommended PA levels. These results may relate to the current predominantly sedentary nature of many occupations and individual difficulties in prioritizing leisure time [39]. Although this finding should be interpreted with caution, as the data do not allow differentiation between discretionary sedentary time and sedentary behavior accumulated during occupational tasks. Given that most participants of the sample were employed and that higher educational achievement is commonly associated with office-based or cognitively demanding professions, the observed pattern may primarily reflect increased occupational sitting rather than lower engagement in PA overall. This interpretation is further supported by the lack of a significant association between educational level and total PA volume [39]. Thus, higher sedentary time among more educated women should not be assumed to indicate poorer health behaviors, but rather differences in work-related activity profiles during pregnancy.

Contrary to expected patterns, pregnant women with higher pre-pregnancy BMI exhibited similarly elevated levels of vigorous PA. This finding contrasts with the results reported by Bernardo et al. [13], who reported that higher pre-pregnancy BMI is associated with lower PA indices during pregnancy. This discrepancy may reflect contextual or behavioral factors not accounted for in the present study. Other studies have documented extremely low levels of vigorous activity in this specific population [25,26,28,40]. However, this result may be explained by Bernardo & Carvalho et al., who highlight the existence of external motivation for pregnant women to control weight gain and/or professional health monitoring that promotes physical activity practice [28]. Nevertheless, this result should be interpreted with caution due to the small contribution of vigorous activity to total PA and the self-reported nature of the data.

These findings highlight the importance of considering social stigma and perceived barriers such as fatigue, lack of time, and discomfort, which have been described in literature as obstacles to PA during pregnancy. Pregnancy has been described in the literature as a potential opportunity for addressing inactivity-related behaviors, rather than as a condition necessarily requiring restriction [13]. Low counseling and literacy among pregnant women, combined with fears regarding potential risks inherent in physical activity, contribute to abandonment or refusal to initiate such practices. Thus, a comprehensive analysis of the effect of PA on musculoskeletal pain control is essential, with strong public health implications [40]. However, the present study was not designed to evaluate the effects of specific interventions.

This study has several limitations that should be acknowledged. The cross-sectional design precludes any inference of causality or temporal relationships between PA patterns and LBP-related disability. Both PA and disability were assessed through self-reported questionnaires (PPAQ and ODI), which, although valid and reliable, remain subject to recall and social desirability biases. The absence of objective assessments of PA and clinical confirmation of LBP may also have influenced the accuracy of the reported measures. Furthermore, the sampling frame is limited to specific regions and healthcare centers and may restrict the generalizability of the findings to the overall population of pregnant women in Portugal. In addition, multiple bivariate analyses were performed, increasing the likelihood of type I errors. The generally modest strength of observed associations further indicates that the results should be considered exploratory and highlights the need for longitudinal and interventional studies to better clarify these relationships. Given that all analyses are unadjusted and cross-sectional, causality cannot be inferred, the potential impact of residual confounding remains, and the findings should, thus, be interpreted with appropriate caution.

The low levels of PA observed in this sample, together with higher disability scores, reinforce the relevance of further research addressing PA patterns and musculoskeletal symptoms during pregnancy. Implementing prevention and education policies within this community to promote increased PA practice may reduce the incidence of such dysfunction.

## 5. Conclusions

Most participants are predominantly engaged in light-intensity or sedentary physical activity, with minimal participation in vigorous activity. In this sample of Portuguese pregnant women, PA levels and types showed weak associated with LBP–related functional disability; however, the observed relationships were generally weak and should be interpreted with caution.

These results highlight the need for further research, particularly longitudinal and interventional studies, to better characterize physical activity patterns during pregnancy and their relationship with low back pain–related disability among the Portuguese population. Although causal inferences cannot be drawn, the results may inform future public health strategies and clinical guidance aimed at supporting safe and appropriate physical activity during pregnancy. Prioritizing the prevention of musculoskeletal dysfunction and challenging entrenched social stereotypes remain essential to improving early primary healthcare interventions in this population.

## Figures and Tables

**Table 1:** Socio-demographic and Obstetric characteristics of the sample.

Socio-Demographic	*n* (%)	Obstetric	*n* (%)
Age (years) (Mean ± SD)	31.7 ± 5.13	Gestational weeks (Mean ± SD)	30.9 ± 6.75
Geographic area *n* ^1^			
North	142 (83.5)	Gestational trimester	
Center	11 (6.5)	1st trimester	4 (2.1)
South	16 (9.4)	2nd trimester	36 (18.8)
Islands	1 (0.6)	3rd trimester	152 (79.1)
Educational level			
Up to 4th grade	1 (0.5)	Parity ^3^	
5th–9th grade	8 (4.2)	Nulliparous	92 (62.6)
10th–12th grade	78 (40.6)	Multiparous	55 (37.4)
Bachelor’s degree	87 (45.3)		
Master’s degree	18 (9.4)	Pre-pregnancy BMI (kg/m^2^) (Mean ± SD)	24.7 ± 4.67
Employment status		Underweight	5 (2.6)
Employee (others)	159 (82.8)	Normal weight	119 (62)
Self-employed	15 (7.8)	Overweight	40 (20.8)
Housewife	3 (1.6)	Obesity	28 (14.6)
Unemployed	14 (7.3)		
Student	1 (0.5)		
Monthly income ^2^			
Up to €705	30 (24.5)		
€705–1000	33 (19.1)		
€1000–1500	43 (22.9)		
€1500–2000	36 (19.1)		
Over €2000	46 (24.5)		
Marital status			
Married/cohabiting	160 (83.3)		
Single	30 (15.7)		
Separated/divorced	2 (1)		

Missing data: 22 responses for the variable. ^1^ Geographic Area = 22; ^2^ Monthly Income = 4; ^3^ Parity = 45. Percentages were calculated using the total number of valid responses as the denominator and are expressed as proportions (%).

**Table 2:** Levels of Intensity and Types of Physical Activity.

	Mean ± SD	% Contribution
Intensity of PA (MET·h·wk^−1^)		
Total	213.9 ± 103.01	100
Sedentary	52.4 ± 28.41	24.5
Light	95.4 ± 45.56	44.6
Moderate	64.3 ± 68.78	30.1
Vigorous	1.8 ± 3.87	0.8
Type of PA (MET·h·wk^−1^)		
Domestic	111.8 ± 61.41	52.3
Occupational	35.9 ± 73.82	16.8
Sports/Exercise	10.4 ± 9.83	4.9
Transportation	18.5 ± 17.62	8.6
Inactivity	37.3 ± 22.35	17.4

**Table 3:** Low back pain and disability according to the ODI score.

Characteristics	Results
ODI Total (mean ± SD)	11.5 ± 7.35
No disability n (%)	6 (3.1)
Minimal disability n (%)	83 (43.2)
Moderate disability n (%)	81 (42.2)
Severe disability n (%)	21 (11.0)
Very severe disability n (%)	1 (0.5)
Symptom exaggeration n (%)	0 (0)

**Table 4:** Correlation between socio-demographic variables, physical activity, and ODI.

Variables	Age		Pre-pregnancy BMI		Gestational weeks		Parity	Educational Level
	**r**	***p* ^1,2^**	**r**	***p* ^1,2^**	**r**	***p* ^1,2^**	***p* ^1,3^**	***p* ^1,3^**
Total PA (MET·h·wk^−1^)	−0.065	>0.371	−0.064	>0.381	−0.103	>0.157	0.005	>0.482
PA Intensity (MET·h·wk^−1^)								
Sedentary	0.043	>0.556	−0.026	>0.722	−0.094	>0.194	>0.740	<0.037
Light	0.062	>0.396	−0.062	>0.392	−0.009	>0.898	<0.001	>0.670
Moderate	−0.139	<0.056	−0.059	>0.418	−0.103	>0.157	<0.045	>0.793
Vigorous	−0.041	>0.579	0.157	<0.029	−0.104	>0.150	>0.819	>0.452
PA Activity (MET·h·wk^−1^)								
Domestic	0.089	>0.223	−0.090	>0.212	0.146	<0.044	<0.001	>0.482
Occupational	−0.086	>0.238	−0.030	>0.676	−0.295	<0.001	>0.571	>0.216
Sports/Exercise	−0.099	>0.176	0.048	>0.512	−0.035	>0.625	>0.097	>0.157
Transportation	−0.100	>0.171	0.047	>0.520	−0.011	>0.876	>0.705	>0.913

PA = Physical Activity; ODI = Oswestry Disability Index; BMI = Body Mass Index; ^1^ *p* < 0.05 is considered indicative of a statistically significant relationship; ^2^ r = Pearson correlation coefficient; ^3^ Kruskal–Wallis test.

**Table 5:** Correlation between levels and types of Physical Activity and Oswestry Disability Index total score.

Oswestry Disability Index	r	*p*
PPAQ Total	0.027	>0.713
PA Intensity (MET·h·wk^−1^)		
Sedentary	0.112	>0.124
Light	0.093	>0.199
Light + Sedentary	0.144	<0.047
Moderate	−0.071	>0.329
Vigorous	0.056	>0.441
PA Activity (MET·h·wk^−1^)		
Domestic	0.103	>0.156
Occupational	−0.082	>0.256
Sports/Exercise	−0.075	>0.301
Transportation	−0.058	>0.427
Inactivity	0.192	<0.008

## Data Availability

All data generated or analyzed during this study are included in this paper. Further inquiries can be directed to the corresponding author.

## Supplementary Materials

The following supporting information can be downloaded at: https://www.mdpi.com/article/10.3390/ijerph23020245/s1, Figure S1. Sensitivity analysis comparing Pearson and Spearman correlation coefficients for variables violating normality assumptions.

## Abbreviations

The following abbreviations are used in this manuscript:

LBP	Low Back Pain
PA	Physical Activity
DLAs	Daily life activities
BMI	Body Mass Index
ACeS	Primary Health Care Units in Portugal and the Autonomous Regions (Azores and Madeira)
CHUSJ	São João University Hospital Center
PPAQ	Pregnancy Physical Activity Questionnaire
ODI—V2	The Oswestry Disability Index

## Appendix A

**Table A1:** Relationship between disability levels according to the ODI score and total physical activity level (PPAQ).

ODI—Disability Levels	r	*p*
Mild disability	0.014	>0.902
Moderate disability	0.068	>0.548
Severe disability	0.155	>0.503

ODI = Oswestry Disability Index; PPAQ = Pregnancy Physical Activity Questionnaire; r = Pearson correlation coefficient. No statistically significant correlations were found (*p* > 0.05).

**Table A2:** Relationship between disability levels according to the ODI score and physical activity intensity levels (PPAQ).

ODI—Disability Levels	Sedentary		Light		Moderate		Vigorous	
	r	*p*	r	*p*	r	*p*	r	*p*
Mild disability	−0.034	>0.764	0.069	>0.534	−0.008	>0.940	0.017	>0.877
Moderate disability	0.023	>0.841	0.152	>0.176	−0.016	>0.888	−0.048	>0.672
Severe disability	0.529	<0.014	−0.069	>0.767	−0.055	>0.813	0.101	0.663

PPAQ = Pregnancy Physical Activity Questionnaire; r = Pearson correlation coefficient. *p* < 0.05 was considered statistically significant.

**Table A3:** Relationship between disability levels according to the ODI score and physical activity types (PPAQ).

ODI—Disability Levels	Domestic		Occupational		Sports/Exercise		Transportation		Inactivity	
	r	*p*	r	*p*	r	*p*	r	*p*	r	*p*
Mild disability	−0.098	>0.378	0.117	>0.292	−0.087	>0.432	0.012	>0.912	−0.104	>0.350
Moderate disability	0.120	>0.286	−0.014	>0.898	−0.071	>0.532	0.060	>0.596	−0.080	>0.946
Severe disability	−0.169	>0.464	0.195	>0.398	0.002	>0.995	0.105	>0.650	0.498	<0.022

PPAQ = Pregnancy Physical Activity Questionnaire; r = Pearson correlation coefficient. *p* < 0.05 was considered statistically significant.
