# Supplementary material for: Physical Activity During Pregnancy: Associations Between Levels and Types of Physical Activity and Low Back Pain–Related Disability in Portuguese Pregnant Women
Source: Int J Environ Res Public Health. 2026 Feb 15;23(2):245. doi: 10.3390/ijerph23020245 (PMC12941212; doi:10.3390/ijerph23020245)
Supplement: Supplementary file 1 [file ijerph-23-00245-s001.zip › ijerph-3996903-supplementary.pdf]

Matriz de Correlações

		O_Idade_anos	O_PPAQ_Sedentária	O_PPAQ_Sdentária_mais_leve	O_Distrito	O_PPAQ_TA	O_PPAQ_Actividade_Moderada	O_PPAQ_Actividade_Vigorosa	O_PPAQ_AC_Domestica	O_PPAQ_AC_Ocupacional	O_PPAQ_ACleve	O_PPAQ_Desporto_Ex	O_PPAQ_Inatividade	O_PPAQ_Deslocacoes	O_PPAQ_TATIPOATV
O_Idade_anos	R de Pearson	—													
	gl	—													
	p-value	—													
	Rho de Spearman	—													
	gl	—													
	p-value	—													
O_PPAQ_Sedentária	R de Pearson	0.043	—												
	gl	187	—												
	p-value	0.556	—												
	Rho de Spearman	0.008	—												
	gl	187	—												
	p-value	0.916	—												
O_PPAQ_Sdentária_mais_leve	R de Pearson	0.062	0.494	—											
	gl	187	190	—											
	p-value	0.396	<.001	—											
	Rho de Spearman	0.065	0.496	—											
	gl	187	190	—											
	p-value	0.377	<.001	—											
O_PPAQ_TA	R de Pearson	-0.065	0.226	0.793	-0.073	—									
	gl	187	190	190	168	—									
	p-value	0.371	0.002	<.001	0.347	—									
	Rho de Spearman	0.006	0.282	0.878	-0.035	—									
	gl	187	190	190	168	—									
	p-value	0.936	<.001	<.001	0.652	—									
O_PPAQ_Actividade_Moderada	R de Pearson	-0.139	-0.030	0.425	-0.075	0.887	—								
	gl	187	190	190	168	190	—								
	p-value	0.056	0.676	<.001	0.332	<.001	—								
	Rho de Spearman	-0.052	-0.123	0.402	-0.050	0.766	—								
	gl	187	190	190	168	190	—								
	p-value	0.481	0.089	<.001	0.521	<.001	—								
O_PPAQ_Actividade_Vigorosa	R de Pearson	-0.041	-0.044	0.081	0.045	0.142	0.093	—							
	gl	187	190	190	168	190	190	—							
	p-value	0.579	0.543	0.265	0.559	0.049	0.198	—							
	Rho de Spearman	-0.044	-0.055	0.036	-0.008	0.101	0.091	—							
	gl	187	190	190	168	190	190	—							
	p-value	0.544	0.447	0.622	0.917	0.161	0.207	—							
O_PPAQ_AC_Domestica	R de Pearson	0.089	-0.063	0.634	-0.073	0.629	0.455	0.126	—						
	gl	187	190	190	168	190	190	190	—						
	p-value	0.223	0.383	<.001	0.345	<.001	<.001	0.083	—						
	Rho de Spearman	0.129	-0.075	0.597	-0.050	0.691	0.598	0.145	—						
	gl	187	190	190	168	190	190	190	—						
	p-value	0.077	0.300	<.001	0.520	<.001	<.001	0.045	—						
O_PPAQ_AC_Ocupacional	R de Pearson	-0.086	0.129	0.364	-0.050	0.674	0.736	-0.028	-0.063	—					
	gl	187	190	190	168	190	190	190	190	—					
	p-value	0.238	0.074	<.001	0.520	<.001	<.001	0.698	0.385	—					
	Rho de Spearman	-0.002	0.198	0.357	-0.033	0.445	0.345	0.021	-0.104	—					
	gl	187	190	190	168	190	190	190	190	—					
	p-value	0.982	0.006	<.001	0.672	<.001	<.001	0.774	0.153	—					
O_PPAQ_ACleve	R de Pearson	0.040	-0.063	0.836	-0.111	0.768	0.507	0.124	0.764	0.334	—				
	gl	187	190	190	168	190	190	190	190	190	—				
	p-value	0.584	0.387	<.001	0.149	<.001	<.001	0.087	<.001	<.001	—				
	Rho de Spearman	0.070	-0.033	0.815	-0.066	0.810	0.508	0.097	0.795	0.211	—				
	gl	187	190	190	168	190	190	190	190	190	—				
	p-value	0.338	0.645	<.001	0.390	<.001	<.001	0.179	<.001	0.003	—				
O_PPAQ_Desporto_Ex	R de Pearson	-0.099	-0.131	0.065	-0.036	0.185	0.185	0.721	0.243	-0.096	0.159	—			
	gl	187	190	190	168	190	190	190	190	190	190	—			
	p-value	0.176	0.071	0.371	0.640	0.010	0.010	<.001	<.001	0.185	0.027	—			
	Rho de Spearman	-0.112	-0.114	-0.014	-0.007	0.103	0.213	0.565	0.177	-0.120	0.085	—			
	gl	187	190	190	168	190	190	190	190	190	190	—			
	p-value	0.126	0.115	0.845	0.926	0.156	0.003	<.001	0.014	0.096	0.240	—			
O_PPAQ_Inatividade	R de Pearson	-0.132	0.643	0.263	0.115	0.081	-0.076	-0.026	0.001	-0.217	-0.101	-0.054	—		
	gl	187	190	190	168	190	190	190	190	190	190	190	—		
	p-value	0.070	<.001	<.001	0.136	0.263	0.295	0.723	0.989	0.002	0.164	0.453	—		
	Rho de Spearman	-0.176	0.674	0.245	0.132	0.057	-0.214	-0.021	-0.036	-0.295	-0.082	-0.015	—		
	gl	187	190	190	168	190	190	190	190	190	190	190	—		
	p-value	0.015	<.001	<.001	0.087	0.434	0.003	0.775	0.620	<.001	0.258	0.832	—		
O_PPAQ_Deslocacoes	R de Pearson	-0.100	0.259	0.535	-0.070	0.623	0.508	0.144	0.322	0.299	0.467	0.150	0.142	—	
	gl	187	190	190	168	190	190	190	190	190	190	190	190	—	
	p-value	0.171	<.001	<.001	0.364	<.001	<.001	0.047	<.001	<.001	<.001	0.037	0.049	—	
	Rho de Spearman	-0.001	0.234	0.525	-0.039	0.575	0.423	0.144	0.346	0.196	0.471	0.190	0.056	—	
	gl	187	190	190	168	190	190	190	190	190	190	190	190	—	
	p-value	0.989	0.001	<.001	0.611	<.001	<.001	0.046	<.001	0.006	<.001	0.008	0.441	—	
O_PPAQ_TATIPOATV	R de Pearson	-0.065	0.226	0.793	-0.073	1.000	0.887	0.142	0.629	0.674	0.768	0.185	0.081	0.623	—
	gl	187	190	190	168	190	190	190	190	190	190	190	190	190	—
	p-value	0.371	0.002	<.001	0.347	<.001	<.001	0.049	<.001	<.001	<.001	0.010	0.263	<.001	—
	Rho de Spearman	0.006	0.282	0.878	-0.035	1.000	0.766	0.101	0.691	0.445	0.810	0.103	0.057	0.575	—
	gl	187	190	190	168	190	190	190	190	190	190	190	190	190	—
	p-value	0.936	<.001	<.001	0.652	<.001	<.001	0.161	<.001	<.001	<.001	0.156	0.434	<.001	—
